# l-Carnitine Supplementation during In Vitro Maturation and In Vitro Culture Does not Affect the Survival Rates after Vitrification and Warming but Alters *Inf-T* and *ptgs2* Gene Expression

**DOI:** 10.3390/ijms21165601

**Published:** 2020-08-05

**Authors:** Diego F. Carrillo-González, Nélida Rodríguez-Osorio, Charles R. Long, Neil A. Vásquez-Araque, Juan G. Maldonado-Estrada

**Affiliations:** 1 One Health and Veterinary Innovative Research and Development (OHVRI) Group, College of Veterinary Medicine, University of Antioquia, Medellin 050034, Colombia; juan.maldonado@udea.edu.co; 2 Faculty of Agricultural Sciences, School of Zootechny, Universidad de Sucre, Sincelejo 700001, Colombia; 3 Genomics and Bioinformatics Lab, Department of Biological Sciences, University of the Republic of Uruguay, Salto Campus, Rivera 1350, Salto 50000, Uruguay; nelida.rodriguez@unorte.edu.uy; 4 Department of Veterinary Physiology and Pharmacology, College of Veterinary Medicine, Texas A&M University, College Station, TX 77843, USA; clong@cvm.tamu.edu; 5 Grupo de investigación Biotecnología Animal, Facultad de Ciencias, Universidad Nacional de Colombia, Sede Medellín, Medellín 050034, Colombia; nvasquez@unal.edu.co

**Keywords:** bovine embryo, cryotolerance, lipid metabolism, gene expression

## Abstract

l-carnitine is a potent antioxidant used for in vitro culture systems. Controversial results have been reported using l-carnitine in culture medium at different stages of in vitro bovine embryo production. Cumulus-oocyte complexes (*n* = 843) were in vitro-fertilized and cultured and added (treatment group) or not added (control group) with l-carnitine. At day three of culture, each group was subdivided into two subgroups receiving no l-carnitine (group 1), 3.8 mM l-carnitine added during in vitro maturation (group 2), 1.5 mM added during the in vitro culture (group 3), and 3.8 mM and 1.5 mM added during the maturation and culture, respectively (group 4). At day 8, blastocyst embryos were examined for mitochondrial activity, the presence of lipid droplets, total cell number, gene expression, and cryotolerance by vitrification. The data were analyzed with a one-way analysis of variance. l-carnitine added in the late in vitro culture significantly reduced mitochondrial activity and lipid content, and upregulated *ifn-τ* and *ptgs2* gene expression compared to controls (*p* < 0.05). l-carnitine supplementation did not significantly affect the embryo rate production or survival rate after vitrification and warming (*p* > 0.05). l-carnitine supplementation significantly improved embryo potential to develop viable pregnancies in agreement with a study reporting improved pregnancy rates.

## 1. Introduction

In all mammalian species, preimplantation embryo development can be divided into two phases based on its metabolic activity: (1) the period controlled by the mRNAs and proteins accumulated in the oocyte and characterized by low metabolic activity, and (2) the period following activation of the embryonic genome, which shows a dramatic increase in metabolism concomitantly with blastocoel cavity formation and expansion. This change in the metabolic activity of the embryo is also associated with the passage of the embryo from the oviduct to the uterus [1]. The knowledge gained for understanding these mechanisms has exerted significant effects on the success rate of in vitro embryo production (IVEP), attributed to the creation of culture media based on metabolic requirements of the embryo and the composition of oviductal and uterine fluids [2,3]. In embryo metabolism, adenosine triphosphate (ATP) synthesis depends on the rate of glucose, pyruvate, lipids, and amino acid consumption [4]. Also, culture media is supplemented with pyruvate and glucose mainly. However, bovine embryos also have glycogen and lipid reserves. The glycogen concentration seems almost negligible and is poorly described in the literature, but lipids (particularly fatty acids) represent the most abundant energy reservoir of bovine embryos. In addition to lipids as a source of energy, which have not been taken into account for the formulation of culture media for in vitro embryo production. Fatty acids are lipid chain biomolecules composed of long-chain hydrocarbons with a terminal carboxyl group, which are found in the cell as energy storage in the form of triacylglycerol (TG) or free fatty acids (FFAs) in the cytoplasm. They can be used as a substrate for the production of phospholipids, and can also serve as an energy source [5]. The FFAs are linked to coenzyme A in the outer mitochondrial membrane, before being transported to the matrix by l-carnitine [5]. Subsequently, it initiates the process of β-oxidation to Acyl-CoA. The cellular fatty acids are stored in lipid droplets composed mainly of triacylglycerols. The oocytes of several mammalian species contain comparatively high amounts of lipids stored as droplets in the ooplasm [5]. Based on oxygen consumption, it has been suggested that there are sufficient lipids to support the metabolic demands for oocyte maturation as the sole source of energy [5]. Lipid metabolism regulators added to culture media can influence the expression of lipid metabolism-regulating genes, and thus, can improve the developmental competence and quality of in vitro produced embryos [6]. l-carnitine is intrinsically involved in mitochondrial function and lipid metabolism via the transport of fatty acids to the mitochondria and its involvement in fatty acid β-oxidation [6]. It also participates in the regulation of cellular functions such as apoptosis [6].

Sprícigo et al. observed that on in vitro maturation (IVM) of porcine oocytes, the translocation of active mitochondria was induced from the periphery of the cytoplasm to the interior or medullar region of the cytoplasm of the oocyte, which favors embryonic development [7]. Similarly, when the media was supplemented with l-carnitine during IVM, the oocyte accelerated its nuclear maturation, increased the number of active mitochondria, and decreased the levels of lipid droplets [7,8]. Likewise, the addition of l-carnitine increased the embryo hatching percentage, an effect attributed to the improvement of embryo quality and its competence for development due to the reduction of lipid content and its developmental competence [8,9]. Moreover, supplementation with l-carnitine has demonstrated to improve the survival rate of embryos subjected to vitrification/heating, an effect related to the variation in the content of lipids present in the embryo [8]. In addition to its metabolic function, l-carnitine has been considered as a potent antioxidant that reduces the accumulation of reactive oxygen species (ROS) and decreases the frequency of apoptosis in animal cells [10]. However, inconsistent results have been reported in terms of embryonic development when vitrified/heated bovine oocytes previously ripened in the presence of l-carnitine have been used [7].

We previously found that l-carnitine used during IVM improved the cumulus cell expansion with no effect on embryo rate on day eight of development [11]. However, embryo quality has not been assessed. With the working hypothesis that l-carnitine could be implicated in the metabolism of bovine embryo cultured in vitro, the aim of this study was to investigate the effect of l-carnitine, supplemented during the in vitro maturation and culture processes, on the embryo quality expressed as the mitochondrial activity, lipid content, total cell number, gene expression, and cryotolerance.

## 2. Results

For testing the effect of l-carnitine on the embryo production rate, a total of 843 oocytes were processed in four independent experiments from IVM until day eight of the in vitro culture. No significant differences (*p* > 0.05) in the cleavage rate at 66 hours post-insemination (hpi) were found between oocytes maturated with l-carnitine and the control group (Table 1). In the same way, early embryo development rate (four cells stage) did not significantly differ (*p* > 0.05) between oocytes maturated with or without l-carnitine (Table 1).

After late in vitro culture, when the l-carnitine (1.5 mM) was added in groups 3 and 4 at 66 hpi, to test the effect on in vitro culture, the proportion of day eight blastocysts did not significantly differ (*p* > 0.05) between all four groups (Table 2.). The effect of l-carnitine supplementation on fatty acid modulation, mitochondrial activity, and total cell number was evaluated through staining methods by observation under fluorescence microscopy. The l-carnitine addition in the culture media during in vitro maturation and late in vitro culture showed that the relative fluorescence in the group 2 (87.30% ± 2.63), group 3 (88.22% ± 3.9), and group 4 (82.61% ± 1.95), were lower than the control group (100% ± 2.56), demonstrating a reduction in mitochondrial activity (Table 2).

On the other hand, when l-carnitine was used as a lipid content modulator in bovine embryos, the supplementation with 3.8 mM of l-carnitine during in vitro maturation (group 2; 88.98 ± 4.2%) had a statistically significant difference (*p* < 0.05) compared to the control group (group 1; 100 ± 3.79%). However, none of the embryos from the treatment group 3 (95.8 ± 2.3%) and group 4 (91.47 ± 2.5%) showed a statistically significant difference (*p* > 0.05) compared to group 1 and group 2 (Table 2).

At day eight post-insemination, the expanded blastocysts from all treatments were stained with Hoechst 33342 to determine the total cell number (TCN) as an indicator of embryo quality indicator. As shown in Table 2, the addition of l-carnitine during IVM (3.8 mM) and IVC (1.5 mM) did not show a statistically significant difference (*p* > 0.05) between groups.

**Figure 1:**
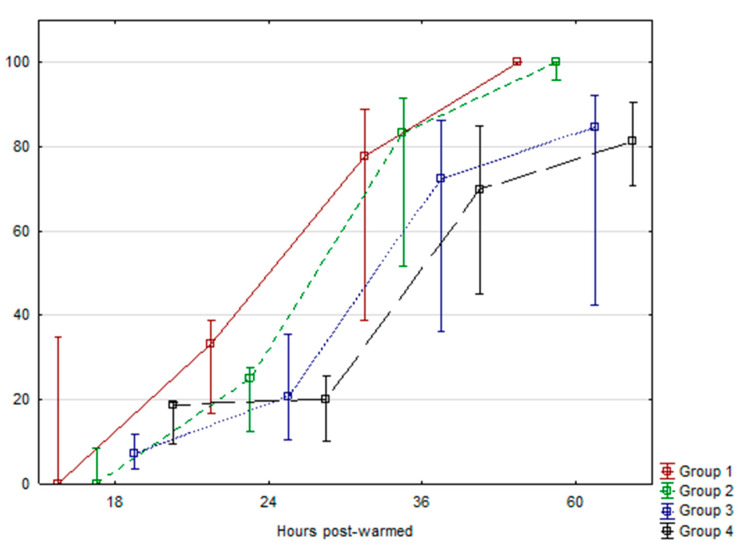
Kinetics of embryo hatching after warming post-vitrification. Data were evaluated using an ANOVA test and Tukey LSD test for comparison between means (*p* < 0.05). Data are expressed as Means ± SEM.

In order to assess the cryotolerance of in vitro produced embryos using l-carnitine, a total of 149 expanded blastocysts from all treatments were subjected to the vitrification process. After at least two weeks in liquid nitrogen, embryos were warmed and subsequently transferred to the incubator in culture media without l-carnitine. The recovery rate was 95.3% (142/149), and the reexpansion processes and hatching process were recorded during the following 18, 24, 36, and 60 h post-warming. The data did not fit a normal distribution. However, the reexpansion after warming was 100% in all treatments and the kinetics of embryonic hatching after warming post-vitrification was recorded (Figure 1). We did not find statistically significant differences (*p* > 0.05) between treatments at any time assessed.

**Figure 2:**
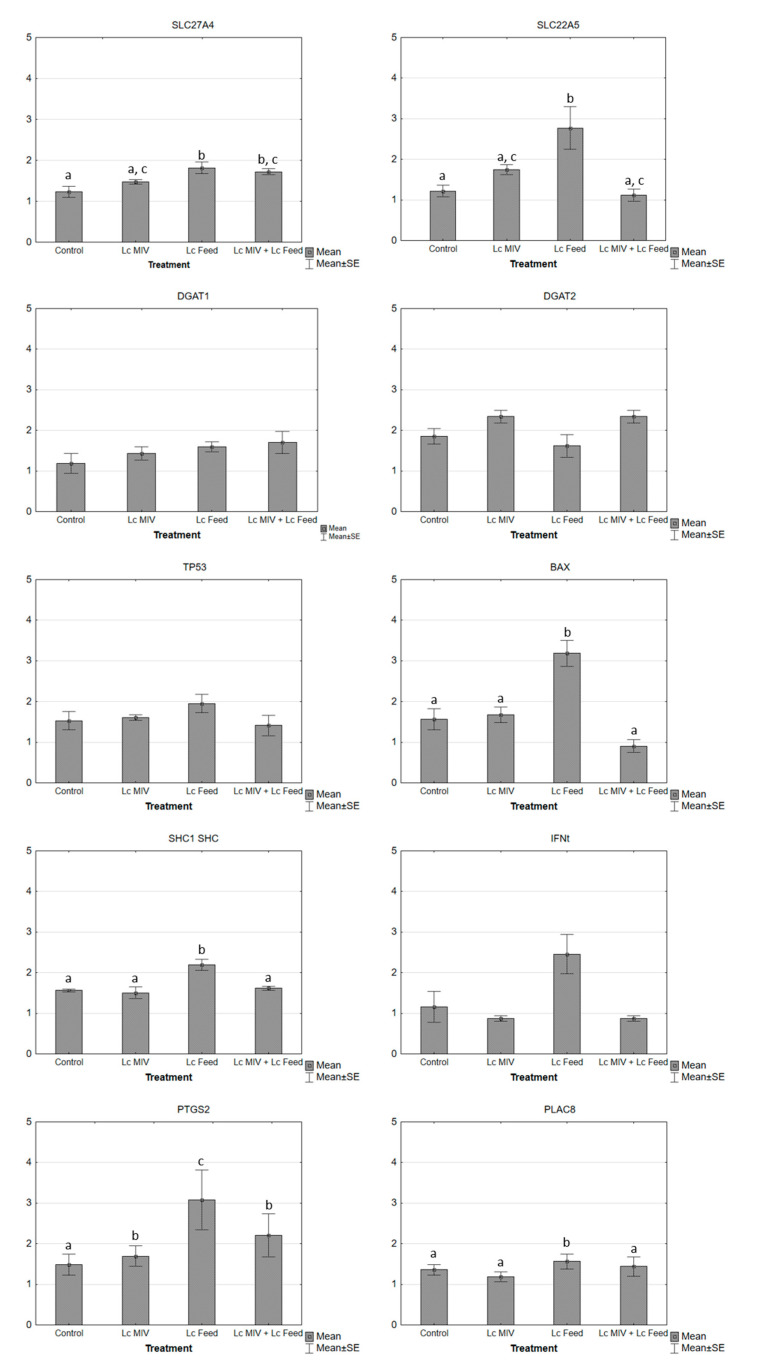
mRNA relative transcript abundance on bovine embryos cultured with l-carnitine at different preimplantation stages using real-time PCR. mRNA relative transcript abundance on bovine embryos cultured with l-carnitine at different preimplantation stages using real-time PCR (mean ± SEM). Means without a common superscript difference (*p* < 0.05).

When the effect of l-carnitine was assessed on the relative expression of transcript candidates, we found that the expression profile for *slc27a4*, *slc22a5*, *shc1*, *shc*, *bax*, *ifn-t*, and *ptgs2* genes significantly increased (*p* < 0.05) in group 3 (embryos cultured with l-carnitine at late IVC) compared to group 1 (control group; Figure 2). Furthermore, in groups 2 and 4, the relative abundance of *slc27a4* and *slc22a5* genes, significantly differed from group 3, but not from group 1. In the case of the *IFN-t* transcript, group 3 had shown a statistically significant difference with all the other experimental groups, and groups 2 and 4 had a difference with group 1. For *dgat1*, *dgat2*, *tp53*, and *plac8*, we did not find a statistically significant difference.

## 3. Discussion

In this work, we showed that l-carnitine added at 3.8 and 1.5 mM during in vitro maturation and in vitro culture of bovine embryos, respectively, reduced the mitochondrial activity in the developed embryos. Also, the addition of 3.8 mM l-carnitine during in vitro maturation significantly affected lipid mobilization. Finally, the addition of l-carnitine in the late culture significantly improved the expression of a group of genes implicated in embryo metabolism such as *slc27a4*, *slc22a5*, *shc1*, *shc*, *bax*, and in maternal recognition of pregnancy, such as the *ifn-t* and *ptgs2* genes, compared to the control group. The expression of other genes significantly varied between groups treated with l-carnitine during in vitro maturation (oocytes) and in vitro culture (developed embryos). l-carnitine has been used in the in vitro embryo production process by its capability to enhance lipid metabolism.

Controversial results are reported on the effect of l-carnitine on cleavage rates of porcine oocytes [12] or the development of porcine embryos [13]. In our work, l-carnitine did not have effect on cleavage rate and the four-cells embryo stage, in agreement with previous reports [6,10,14,15,16,17], despite slight differences in the concentrations used and the development stage evaluated by these authors. Also, our work showed that supplementation with l-carnitine during in vitro maturation, and late in vitro culture did not affect the blastocysts rate, in agreement with the reports by Held-Hoeker et al. (using 2.5 mM of l-carnitine in Synthetic Oviductal Fluid (SOF) media during in vitro culture) [16], Knitlova et al. using 2.5 mM of l-carnitine in the maturation media in meiotically more competent oocytes derived from medium-sized follicles (6–10 mm) [6], and Zolini [17] using 1.5 mM and 3.03 mM of l-carnitine during the embryo culture. In our study, no effect of 1.5 mM l-carnitine was observed in the early embryo development rates during the in vitro culture. Ghanem reported a significant reduction in embryo rate production using 1.5 mM l-carnitine during in vitro culture [15]. Most authors use the l-carnitine during in vitro maturation with no addition during in vitro culture [6,10,14,15,17]. Phongnimitr et al. found that 0.6 mg/mL l-carnitine improved the blastocyst production rate from 23.4 (control group) to 29.7% (l-carnitine group) [14], and Knitlova [6] found that 2.5 mM l-carnitine improved the embryo production rate from meiotically less competent oocytes (33.3%) compared to the control group (25.83%). These studies suggest that l-carnitine can exert beneficial effects on in vitro produced embryos only under specific conditions and quality of oocytes used in the system.

In our study, the mitochondrial activity was significantly reduced (*p* < 0.05) when adding l-carnitine (groups 2, 3, and 4; Table 2). The use of l-carnitine as a modulator and stimulator of mitochondrial activity through the beta-oxidation pathway during oocyte maturation has been previously reported [6,18], but no studies are available on the addition of l-carnitine in the in vitro culture and early embryonic development. Oxidative metabolism is the leading source for ATP in early (pre compaction) embryonic stages, with sodium pyruvate being the preferred tricarboxylic acid substrate [19]. l-carnitine could work as a co-factor in the ATP production (for the β-oxidation process), improving the efficiency of ATP production by competent embryos while rescuing embryos that would otherwise fail to develop [19]. In our work, l-carnitine significantly reduced the mitochondrial activity of the embryos, a finding that could be explained by the Leese “hypothesis”, which states that embryos cultured under the appropriate culture conditions reduce its oxygen consumption until achieving basal metabolism [20]. These embryos could be subjected to less damage to the genome, transcriptome, and proteome, or could be better equipped to deal with damage when it occurs, devoting fewer resources to “running repairs” [20].

In addition, the lipid content was reduced in the blastocysts when l-carnitine (3.8 mM) was added during the in vitro maturation (group 2) in comparison with the control group (group 1; Table 2). This result is similar to that reported by Takahashi et al. who found a reduction in lipid content using 3.03 mM l-carnitine during the in vitro embryo culture [10]. Also, Baldoceda et al. showed that adding 0.5 mM l-carnitine significantly reduced the number of lipid droplets in *Bos taurus* embryos [18]. In our work, we used COCs harvested from ovaries of mixed *Bos indicus* breeds (Zebu cattle) where lipid content has been reported to be lower [21], a fact that could explain the lack of statistically significant differences in fluorescence intensity.

In this study, no effect of l-carnitine was found on the TCN of in vitro produced embryos, in agreement with the reports by Mishra et al. using 10 mM l-carnitine during in vitro maturation [22], and Knitlova et al. using 2.5 mM l-carnitine (C0283) during in vitro maturation in meiotically less competent oocytes derived from small follicles (<5 mm) [6]. In contrast, other researchers found improved TCN when adding l-carnitine during in vitro maturation or in vitro culture [10,14,15,23]. Therefore, it is difficult to conclude whether the l-carnitine added during maturation or in vitro culture may or may not affect the total number of embryonic cells.

Our study showed that l-carnitine did not affect the re-expansion and hatching rates after blastocysts warming, independently of the embryo production stage in which l-carnitine was added. A possible explanation could be the lack of statistically significant differences in the lipid content of the embryos between treatments. Additionally, in the culture media used for the in vitro embryo production, BSA and FBS were added, which have been associated with a reduction of embryonic cryotolerance by stimulating the formation of lipid droplets [24,25,26,27]. Also, in our work, we used l-carnitine at different concentrations, during in vitro maturation, late in vitro culture (after 48 h) together and separately, with no effect on embryonic cryotolerance in agreement with reports by Phongnimitr et al. who used 0.6 mg/mL l-carnitine during maturation [14], Sprícigo et al. where 3.03 mM l-carnitine was added to the in vitro maturation of calf oocytes [7], Saravia et al. where 3.72 mM (0.06 mg/mL) l-carnitine was added (Sigma C0158 inner salt) to the vitrification solutions for bovine embryos [28], and Zolini et al. who used 3.03 mM l-carnitine during in vitro maturation and 0.75, 1.5 and 3.03 mM during the in vitro culture.

In contrast, Takahashi and colleagues found that adding 1.5 or 3.03 mM l-carnitine during in vitro culture improved the survival rates after slow-freezing [10]. Chankitisakul and others showed that adding 3.03 mM l-carnitine during in vitro maturation and later vitrifying the oocytes, significantly improved the embryo production rate, attributed to l-carnitine-mediated lipid modulation [29]. In the study by Ghanem et al., using 1.5 mM l-carnitine during late in vitro culture (eight-cell stage), the survival rate was not affected but the hatching rate was significantly improved [23]. Lowe et al., working with porcine embryos, demonstrated significantly improved post-warming survival rate with 3 mM l-carnitine during in vitro culture depending on the media system used [13]. Held-Hoeker and colleagues showed that 2.5 mM l-carnitine added in SOF medium during in vitro culture significantly improved the post-warming hatching rate of bovine embryos [16]. Verma et al. found that adding 1.5 or 2 mM l-carnitine on mSOF medium (without FBS) during in vitro culture, significantly improved the post-thawing survival rate in buffalo embryos [30]. The discrepancies between these studies in terms of the effects of l-carnitine supplementation on embryonic cryotolerance are likely due to the differences in terms of l-carnitine concentrations, the molecular weight of the l-carnitine used, the culture media used, and the presence or absence of several macromolecules, including FBS and BSA [17].

In this study, we found an upregulation of the relative mRNA abundance of *slc27a4* in groups 3 and 4 compared with the control group. *Slc27a4* is involved in the translocation of long-chain fatty acids through the plasma membrane. Besides, *Slc27a4* plays a fundamental role in the regulation of the substrates of free fatty acids from exogenous sources when there is an increase in beta-oxidation or triglycerides synthesis. The SLC27A4 protein has Acyl-CoA ligase activity for long-chain and very-long-chain fatty acids so that it may participate as a facilitator in beta-oxidation. Our result is in agreement with the report by Ghanem et al. [23]. Similarly, we found a significant increase in the expression of the *slc22a5* gene (a gene encoding the organic cation/carnitine transporter 2 (OCNT2) protein) in embryos added with l-carnitine during in vitro culture (group 3), compared to other l-carnitine treated groups (groups 2 and 4) and the control group (group 1). The increased expression of the *slc22a5* gene is associated with increased production of the OCNT2 protein, which is responsible for transporting l-carnitine from the extracellular medium into the embryonic cells. However, we did not evaluate the expression of OCNT2. We found no effect of l-carnitine on the relative expression of *dgat1* and *dgat2* genes, where, in contrast to the result reported by Ghanem et al. [23], Verma et al. [30] found a significant reduction of *dgat1* and *dgat2* gene expression using l-carnitine.

Apoptosis could be induced via oxidation by ROS generation when oocytes or embryos are cultured in vitro, and l-carnitine reduces oxidative stress-induced apoptosis by increasing intracellular glutathione levels and affecting mitochondrial functions [31]. Thus, we examined the expression of *bax*, *tp53*, and *shc1shc* (P66^shc^) genes related to apoptosis, and found that l-carnitine significantly upregulated the expression of the stress sensor gen *p66^shc^* and the pro-apoptotic *bax* gene, in agreement with the report by You and colleagues using 10 mM l-carnitine on pig embryos [31]. This result is contrary to previous reports [10,22,30]. The *p66^Shc^* is involved in signaling pathways that regulate the cellular response to oxidative stress and life span [32]. Also, *p66^Shc^* acts as a downstream target of the tumor suppressor p53 and is essential for the ability of stress-activated p53 to trigger intracellular ROS generation and apoptosis [32], and regulates intracellular ROS-induced DNA damage leading to permanent embryo arrest and apoptosis [32]. We concluded that *bax* expression may not be a good indicator to predict the developmental competence of oocytes and embryos as supported by You et al. [31].

Interferon tau (IFN-t) is one of the genes mostly associated with the quality of in vitro produced embryos; it codes for a protein transcribed from multiple genes (there are at least 12 variants of cDNA) in trophoblast cells [33]. IFN-t is the molecule responsible for maternal recognition of pregnancy in ruminants, a process that prevents luteolysis from occurring. In addition to its anti-luteolytic effect, the paracrine effects of IFN-t promote embryonic development and endometrial receptivity by stimulating the expression of genes related to elongation and implantation in the endometrium [34]. In this work, we demonstrated that adding l-carnitine significantly upregulates the expression of INF-t during the in vitro culture, in agreement with other reports [15,23,30]. Accordingly, it can be argued that blastocysts cultured in the presence of l-carnitine may most likely be able to survive after the transfer, by increasing INF-t production as previously reported [23].

In the same way, Prostaglandin-endoperoxide synthase 2 (PTGS2; also known as Cyclooxygenase 2 (COX2), is the rate-limiting enzyme in the synthesis of prostaglandins (PGs), which are one of the most important mediators of embryo-uterine communication at the initiation of implantation [35]. We observed a higher expression of PTGS2 in group 3. In bovine embryos, the PTGS2 protein is located in trophectoderm cells. The higher expression observed in group 3 could be due to an altered proportion of inner cell mass to trophectoderm cells [36]. However, the expression of PTGS2 is more abundant in blastocysts resulting in a live-born calf compared with those resulting in resorption. PTGS2 gene expression increased throughout the implantation window, which indicates an essential role in prostaglandins released by the embryo in mediating interactions with the uterus [37]. On the other hand, a direct effect between the l-carnitine supplementation and *inf-t* and *ptgs2* gene expression is unknown. However, the upregulation of these genes could be an indicator of an improvement in embryo quality when the l-carnitine is used during late in vitro culture without affecting the embryo production rate.

In summary, 1.5 mM of l-carnitine can be used as a supplement in the in vitro embryo production as a metabolism modulator; it appears to improve the embryos’ “quiet” metabolism and expression of genes such as *inf-t* and *ptgs2* associated with better embryo quality. Moreover, post-thaw cryotolerance in vitro was not affected by l-carnitine supplementation during maturation nor by supplementation during culture [17]. Further research could be indicated to assess the effect of the l-carnitine during the addition of in vitro culture medium in the pregnancy rate after embryo transfer of in vitro-produced embryos.

## 4. Materials and Methods

The Institutional Board on Animal Subject Experimentation from the University of Antioquia approved this work (act 115 of 6 February 2018).

### 4.1. Experimental Design

l-carnitine was added at different preimplantation stages, 3.8 mM was used on in vitro maturation [10,14] and 1.5 mM on late in vitro culture [15]. Embryos from the control group (untreated) were not supplemented with l-carnitine. l-carnitine (l-carnitine inner salts, Sigma-Aldrich St Louis, MO, USA, Product number C0158) was selected according to our previous results [11,38]. Cumulus-oocyte complexes (COCs) were randomly allocated to either the control group (No addition of l-carnitine) or the treatment group, which received 3.8 mM l-carnitine. Afterward, oocytes were in vitro fertilized and cultured. Furthermore, in the late in vitro culture (day 3, from four cells stage until day 8 blastocyst), each group from the in vitro maturation groups was separated into two new groups for a total of four groups: group 1, control group (non l-carnitine supplemented); group 2, l-carnitine added during in vitro maturation (3.8 mM); group 3, l-carnitine added during the in vitro culture (1.5 mM), and group 4, l-carnitine added during maturation and culture (3.8 mM, and 1.5 mM respectively).

### 4.2. Oocyte Collection

Bovine ovaries were obtained post-mortem from cycling cows (as evidenced by the presence of corpus luteum) at a slaughterhouse. The recovered ovaries were placed in bags with sterile saline phosphate buffer solution (PBS) at 35 °C and were transported (approximately within 30 min) to the laboratory for processing. In the laboratory, under sterile conditions, the ovaries were washed three times with 1× PBS at 35 °C to remove contaminating material, blood, and tissue debris. Then with a 10 mL syringe using 18 G needles, follicles with a diameter of 3 to 6 mm were aspirated. The follicular fluid was collected in 50 mL conical tubes placed in a water bath at 35 °C. Subsequently, follicular aspirates were filtered using a 70 μM pore diameter filter and oocytes from were placed in a 60 × 15 mm sterile petri dish containing 3 mL of 1× PBS. Good quality cumulus-oocyte complex (COCs) were selected according to previously established criteria [39,40], under high stereomicroscope vision. Selected COCs were washed three times in Medium 199, Hanks’ Balanced Salts (Gibco™ Gaithersburg, MD, USA, Catalog number: 12350039) before their transfer to the maturation medium.

### 4.3. Oocyte Maturation

COCs (*n* = 10/each treatment) were placed in drops of 50 µL of maturation Medium 199, Hanks’ Balanced Salts (Gibco™, Catalog number: 12350039), containing 25 mM l-glutamine, 6 mg/mL Fraction V Albumin (BSA), 3% FBS, 25 µg/mL FSH (Folltropin), 5 IU/mL LH (Chorulon), 1 µg/mL 17-beta estradiol, 0.30 mM sodium pyruvate and 100 µM ascorbic acid, and covered with mineral oil in a 35 mm culture dish. COCs were randomly allocated to either the control group or the treatment group, which received 3.8 mM l-carnitine supplementation [11,14]. Each group was incubated at 38.5 °C, 5% CO_2_ in the air with 90% relative humidity for 24 h.

### 4.4. Oocyte Fertilization

In vitro-matured COCs were fertilized with frozen-thawed bovine sperm from a batch that had previously been used in our laboratory. COCs (10/drop) were transferred to 50 μL FERT-TALP medium (IVL02, Caisson Laboratories, 836 South 100 East Smithfield, UT 84335) supplemented with 0.1 mg/mL Heparin (Sigma H0519), 1 mM Hypotaurine (Sigma H1384), 250 mM Epinephrine (Sigma E4642), 2 mM Penicillamine (Sigma P4875), and 1x antibiotic solution (ICN 1670049 MP Biomedicals, Fisher Scientific Company, Ottawa, ON, USA). Semen was thawed at 36 °C for 1 min, and motile spermatozoa were separated using percoll gradients (Allgrad^®^ 90% and 45% LifeGlobal group, 393 Soundview Rd, Guilford, CT 06437) by centrifugation at 780× *g* for 5 min at room temperature. The pellet was diluted with 300 μL of FERT-TL media, and the final concentration was adjusted at 2 × 10^6^ sperm/mL, and added in each drop containing COCs [11]. COCs and semen were incubated for 18 to 20 h at 38.5 °C in a humidified atmosphere of 5% CO_2_ in air [23,41].

### 4.5. Assessment of Embryo Culture and Development

At 18 hpi, presumptive zygotes were stripped from cumulus cells and cultured (10/drop) in drops of 70 μL of Synthetic Oviductal Fluid (SOF; IVL05, Caisson Laboratories, 836 South 100 East Smithfield, UT 84335) supplemented with 6 mg/ml BSA-FAF, 1× antibiotic solution, and 3% FBS, at 38.5 °C, 5% CO_2_ and 90% relative humidity. At 48 h after the initiation of culture, the cleavage rate was determined from the total number of oocytes placed for insemination. To evaluate the effect of l-carnitine during late IVC (66 hpi), embryos were divided into four groups, group 3 and group 4 were supplemented with l-carnitine (1.5 mM) until day 8 [10,23]. The in vitro embryo production rate on day eight was determined based on the total number of inseminated oocytes [42].

### 4.6. Determination of Mitochondrial Activity in Embryos Cultured in the Presence of l-Carnitine

The embryos’ mitochondrial activity was assessed with Mitotracker green fluorescent dye. All groups of embryos (blastocyst and expanded blastocyst) were incubated at room temperature for 30 min in Dulbecco’s PBS with 125 nM Mitotracker, according to the manufacturer’s recommendations [23]. After this time, the excess dye was removed by washing twice with Dulbecco’s PBS. Embryos were passed in groups of five structures to 10 μL Dulbecco’s PBS drops on a slide [43]. For its lecture, a B-2E/C filter was used at 480 nm and 520 nm wavelength excitation emissions, respectively. Images were recorded in *.TIF format and the results of the mean fluorescence intensity (MFI) were assessed using the ImageJ software (Version 1.41; National Institutes of Health, Bethesda, MD, USA). The MFI of embryos in the control group was used as a normalizer for the fluorescence intensity measurement of embryos in the l-carnitine groups [44].

### 4.7. Determination of Lipid Droplets in Embryos Cultured in the Presence of l-Carnitine

In order to determine the content of lipid droplets, blastocysts, and the expanded blastocyst of each group were stained with Nile Red. First, embryos were incubated at 4 °C for 40 min in a 1.5 mL conical tube containing 250 μL of fixation medium (1× Dulbecco’s PBS, 3.7% formaldehyde and 3 mg/mL of polyvinylpyrrolidone). After incubation, embryos were washed twice in Dulbecco’s PBS and passed to a 1.5 mL tube with 250 μL of Nile red solution (10 μg/mL) at room temperature for 4 h [45]. Subsequently, the excess dye was removed by washing twice with Dulbecco’s PBS, and embryos were placed on a slide in a 10 μL Dulbecco’s PBS drop [43]. They were observed under a 20× objective using an epifluorescence microscope (Nikon Eclipse 80i, Melville, NY, USA) with a G-2A filter at an excitation wavelength of 510–560 nm and 580 nm emission. The embryo area was adjusted, and images were recorded in *.TIF format. The fluorescence intensity of the lipid droplets in the embryos was analyzed using the ImageJ software (Version 1.41; National Institutes of Health, Bethesda, MD, USA). The MFI of the embryos in the control group was used as a normalizer for the MFI measurement of embryos in the l-carnitine groups [44].

### 4.8. Quantification of Cellularity in Bovine Embryos Cultured in the Presence of l-Carnitine

In order to evaluate the effect of l-carnitine supplementation during oocytes in vitro maturation and in vitro early development processes on embryo cellularity, we used staining with Hoechst 33342 [46]. Expanded blastocysts obtained in each treatment were incubated in the dark for 10 s in PBS with 0.2% (*v*/*v*) Triton 100X, BSA (2 mg/mL). Later, embryos were washed twice and stained with Hoechst 33342 (H1399 ThermoFisher; 10 μg/mL) for 30 min at room temperature. The embryos were washed twice with PBS-BSA and transferred to a 10 μL drop on a slide and covered with coverslips with a little drop of Vaseline in the corners, adding pressure until the pellucid zone was broken [46]. The cell count was performed in the ImageJ program version 1.41, on a digital photograph obtained from a microscope (Nikon^®^ eclipse 80i, Melville, NY, USA). The blue cells were visualized under the UV2E fluorescence filter with a 360 nm excitation length and emission at 460 nm. The total cell number was determined [46,47].

### 4.9. Vitrification, Warming, and Subsequent Embryo Culture

A total number of 149 embryos were vitrified by using the Cryotech vitrification kit 101 (Reprolife, Tokyo, Japan). Five replicates were conducted. Expanded blastocysts were used to the vitrification process following the manufacturer’s instructions. Briefly, the embryos were transferred to the equilibration solution (ES) for 5 min at room temperature. Later, the embryos were transferred to tge vitrification solution (VS) and left there for up to 60 s. Subsequently, pools of five embryos from the same treatment were put in the cryotec support and immediately submerged into liquid nitrogen. Afterward, the cap was placed and stored in a nitrogen tank until their future warming [48].

For warming, the embryos were warmed using the Cryotech warming kit 102 (Reprolife, Tokyo, Japan). Quickly, after removal of the cap, each Cryotop strip that contained the embryos, was placed in a warming solution (TS) drop of 100 µL and waited for 1 min. After that time, embryos were transferred to the diluent solution (DS) and waited for 3 min. Then, embryos were washed twice in washing solution (WS), waiting 5 min each time. Later, each embryos group was transferred to the SOF medium drops (70 µL) and were cultured for 60 h for their posterior re-expansion and hatching assessment [48]. Furthermore, the percentages of the embryos that resumed their development and reached a more advanced developmental stage after culture (development rate), as well as the hatching rates, were recorded [49].

### 4.10. RNA Extraction, Reverse Transcription, and Quantification of mRNA Transcript Abundance

Relative mRNA abundance analysis was performed in three biological replicates, each containing 10 expanding blastocysts per experimental group obtained on day 7 and day 8 post-fertilization, using the Dynabeads^®^ mRNA DIRECT™ Micro Kit (Ambion^®^, Thermo Fisher Scientific Inc., Oslo, Norway), following the manufacturer’s instructions with minor modifications [23,50,51]. After 10 min of incubation in lysis buffer with Dynabeads, poly (A) RNA attached to the Dynabeads was extracted with a magnet and washed twice in washing buffer A, and washing buffer B. RNA was eluted with Tris-HCl. Immediately after extraction, the RT reaction was carried out with RevertAid First Strand cDNA Synthesis Kit (K1622, Thermo Fisher Scientific Inc., Oslo, Norway). Using poly (T) primers, random primers, and Moloney Murine Leukemia Virus (MMLV) High-Performance Reverse Transcriptase enzyme, mixed in a total volume of 40 µL, in order to prime the RT reaction and produce cDNA. Tubes were heated to 70 °C for 5 min to denature the secondary RNA structure, and then the RT mix was completed with the addition of 50 IU of reverse transcriptase. Next, the tubes were incubated at 25 °C for 5 min to promote the annealing of random primers, for 60 min at 42 °C to allow the RT of RNA, and finally at 70 °C for 5 min to denature the enzyme.

All mRNA transcripts were quantified using quantitative polymerase chain reaction (qPCR) and were run in the following conditions: 95 °C for 3 min, 35 cycles of 94 °C for 15 s, 56 °C for 30 s, and 72 °C for 15 s, followed by a final extension step for 10 s. Two replicates for all genes of interest were performed in the Rotorgene 6000 Real-Time Cycler TM (Corbett Research, Sydney, Australia) by adding a 2 mL aliquot of each sample to the PCR mix (QuantiTect SYBR Green PCR Kit, Qiagen, Germantown, MD, USA) containing the specific primers selected to amplify the genes of interest. The comparative cycle threshold (CT) method was used to quantify expression levels. The values were normalized to the endogenous controls (housekeeping gene—histone H2AFZ) [52,53]. According to the comparative CT method, the ΔCT value was determined by subtracting the HK mean CT value for each sample from each gene CT value of the sample. The calculation of ΔΔCT involved using the highest treatment ΔCT value, i.e., the treatment with the lowest target expression as an arbitrary constant to subtract from all other ΔCT sample values. Fold changes in the relative gene expression of the target were determined using the 2^−ΔΔCT^ formula [54]. The primers used for the qPCR were as follows—H2A histone family, member Z (*h2afz*), long-chain fatty acid transport protein 4 (*slc27a4*), membrane transport protein associated with l-carnitine deficiency (*slc22a5*), Tumor protein p53 (*tp53*), Bcl-2-associated X protein (*bax*), SHC transformant protein 1 (*shc1 shc*), interferon tau (*ifn-t*), prostaglandin-endoperoxide synthase 2 (*ptgs2*), Placenta-specific gene 8 protein (*plac8*), and Diacyl glycerol transferase 1 and 2 (*dgat1* and *dgat2*). The primer sequences and the approximate sizes of the amplified fragments of all the transcripts are shown in Table 3.

### 4.11. Statistical Analysis

Data from all experiments were performed at least in three independent replicates for each treatment, and at least 15 embryos from each treatment were analyzed with one-way analysis of variance (ANOVA), using the general linear model (GLM) procedure. The results were expressed as mean ± SEM. All percentage data were arcsine-transformed before analysis. Data on the embryo production rate, mitochondrial activity, relative lipid content, total cell number, cryotolerance, and relative mRNA abundance were analyzed using an ANOVA, with a statistically significant value considered at *p* < 0.05. The differences between the treatments were compared using the Tukey LSD test. The program used was Statistica, version 10.0 (StatSoft Inc. Tulsa, OK, USA).

## Figures and Tables

**Table 1:** Effect of l-carnitine supplementation on cleavage and four-stage cells of in vitro produced bovine embryos.

IVM Treatment	Total Oocytes Maturated	Cleavage Rate		4-Cells Stage	
		*n*	Means ± SD	*n*	Means ± SD
**Control**	431	363	84.09 ± 9.44	318	73.05 ± 12.05
l-carnitine (3.8 mM)	412	342	82.65 ± 4.60	270	65.34 ± 9.75

Data were evaluated using ANOVA test and Tukey’s test for comparison between means (*p* < 0.05). Data are expressed as Means ± SD. Abbreviations: *n* = number, SD = Standard deviation, IVM = In vitro maturation.

**Table 2:** Effect of l-carnitine supplementation on embryo development rate, mitochondrial activity, relative lipid content, and total cell number of in vitro produced bovine embryos.

Treatment	Blastocyst Rates			Mitochondrial Activity			Relative Lipid Content			TCN		
	*n*	Means	±SEM	*n*	Means	±SEM	*n*	Means	±SEM	*n*	Means	±SEM
Group 1	42	26.60	5.86	37	100 ^a^	2.56	30	100 ^a^	3.79	18	133	8
Group 2	54	26.51	4.09	36	87.30 ^b^	2.63	24	88.98 ^b,c^	4.25	17	124	8
Group 3	73	27.41	3.08	28	88.22 ^b^	3.90	41	95.84 ^a,c^	2.38	28	132	6
Group 4	62	29.47	2.82	42	82.61 ^b^	1.95	38	91.47 ^a,c^	2.56	26	138	4

Data were evaluated using the ANOVA test and Tukey LSD test for comparison between means (*p* < 0.05). Data are expressed as Means ± SEM. ^a,b,c^ Values with different superscripts are significantly different *p* < 0.05. Abbreviations: *n* = number, SEM = Standard error of the mean, TCN = Total Cell Number.

**Table 3:** Details of primers used for quantitative real-time PCR analysis.

Gene	Primer Sequence (5′−3′)	Fragment Size (bp)	GenBank Access No.
*slc27a4*	F: 5-ACTGTCAGGCGTGATATCTT-3	203	NM_001075667
	R: 5-AGATCAGGGCTGTCTTGTC-3		
*slc22a5*	F: 5-ACATCTACCTGTCCACCATC-3	173	NM_001046502
	R: 5-CCTACAAGGAAAAACAGCAC-3		
*tp53*	F: CTCAGTCCTCTGCCATACTA	364	NM_174201.2
	R: GGATCCAGGATAAGGTGAGC		
*bax*	F: CTACTTTGCCAGCAAACTGG	158	NM_173894.1
	R: TCCCAAAGTAGGAGAGGA		
*shc1 shc*	F: GGTTCGGACAAAGGATCACC	335	NM_001075305.1 (NM_001075305.2 updated)
	R: GTGAGGTCTGGGGAGAAGC		
*ifn-t*	F: 5-CTGGGAAATCATCAGAGTGGAG-3	279	NM_001015511.3
	R: 5-TAAGGACTCATGCCCCTACAG -3		
*ptgs2*	F: ATCTACCCGCCTCATGTTCCT	187	NM_174445.2
	R: GGATTAGCCTGCTTGTCTGGA		
*plac8*	F: CGGTGTTCCAGAGGTTTTTCC	166	NM_001025325.2
	R: AAGATGCCAGTCTGCCAGTCA		
*dgat1*	F: 5-TCCACTCCTGCCTGAACGC-3	165	NM_174693
	R: 5-GCTGCCCACTTGCTGCTG-3		
*dgat2*	F: 5-GCTTGACTGCAGGACTAAAC-3	151	NM_205793
	R: 5-GCTCAGATTTCAGAGACTGG-3		
*h2afz*	F: 5-AGGACGACTAGCCATGGACGTGTG- 3	208	NM_174809
	R: 5-CCACCACCAGCAATTGTAGCCTTG-3		

Abbreviations: PCR, polymerase chain reaction; bp, base pair.
